# Genetics in psychiatry: Methods, clinical applications and future perspectives

**DOI:** 10.1002/pcn5.6

**Published:** 2022-04-07

**Authors:** Chiara Fabbri

**Affiliations:** ^1^ Department of Biomedical and Neuromotor Sciences University of Bologna Bologna Italy; ^2^ Institute of Psychiatry, Psychology & Neuroscience King's College London London UK

**Keywords:** gene, genetics, GWAS, polygenic risk score, psychiatry

## Abstract

Psychiatric disorders and related traits have a demonstrated genetic component, with heritability estimated by twin studies generally between 80% and 40%. Their pathogenesis is complex and multi‐determined: environmental factors interact with a polygenic architecture, making difficult the development of models able to stratify patients or predict mental health outcomes. Despite this difficult challenge, relevant progress has been made in the field of psychiatric genetics in recent years. This review aims to present the main current methods in psychiatric genetics, their output, limitations, clinical applications, and possible future developments. Genome‐wide association studies (GWASs) performed in increasingly large samples have led to the identification of replicated genetic loci associated with the risk of major psychiatric disorders, including schizophrenia and mood disorders. Statistical and biological approaches have been developed to improve our understanding of the etiopathogenetic mechanisms behind genome‐wide significant associations, as well as for estimating the cumulative effect of risk variants at the individual level and the genetic overlap between different disorders, as pleiotropy is the rule rather than the exception. Clinical applications are available in the pharmacogenetics field. The main issues that remain to be addressed include improving ethnic diversity in genetic studies and the optimization of statistical power through methodological improvements, such as the definition of dimensional phenotypes with specific biological correlates and the integration of different types of omics data.

## INTRODUCTION

Psychiatric disorders are complex diseases with a genetic–environmental pathogenesis. Using a simple metaphor, this etiopathogenetic model can be described as a jar: individuals are born with a certain amount of genetic risk factors that fill the jar until a certain level, as psychiatric disorders are polygenic, that is, many variants spread across the genome are involved in disease susceptibility. This genetic architecture is due to negative selection that leads to the purging of large‐effect mutations in critical regions.^1^ With time, environmental risk factors may accumulate, and increase the level the jar is filled to, making the individual more vulnerable, until a certain threshold value is reached and the individual manifests an active episode of illness.^2^ The degree of contribution from genetic variants is variable for different psychiatric disorders, as family studies have shown, for example a relative risk (RR) of 1.5 in first‐degree relatives of patients with major depressive disorder (MDD) compared to the general population, while an RR of 7 for schizophrenia (SCZ) and bipolar disorder (BP), and almost 9 for autism spectrum disorders (ASDs).^3^

As family studies have suggested a clear genetic component to psychiatric illness, specific approaches have been developed to quantitatively estimate heritability, that is, the proportion of variance in a disease that is attributable to the effect of genetic variants. Twin studies estimate heritability by modelling the variance of phenotypic concordance across monozygotic and dizygotic twin pairs. They have confirmed that psychiatric disorders are heritable, with heritability of about 80% for SCZ and BP, while around 40%–50% for MDD.^4^, ^5^, ^6^ Twin studies do not identify the specific genomic regions/loci involved; that is the main aim of genome‐wide association studies (GWASs).

GWASs analyze the effect of millions of common genetic variants throughout the genome. Typically, several hundreds of thousands are genotyped using a high‐throughput technology and then genotyped variants are imputed to increase their number up to ~10 million, by exploiting known relationships among variants (linkage disequilibrium [LD]) and reference panels obtained in samples of the same ethnic group. As GWASs include only common genetic variants (usually defined as those with a frequency ≥1% in the population), mostly single nucleotide polymorphisms (SNPs), heritability estimated using GWASs is typically half or less compared to heritability estimated by twin studies, for example, about 30% for SCZ, and this discrepancy is referred to as missing heritability.^5^ Rare variants, such as deletions and duplications (copy number variants), may explain a relevant part of the missing heritability.

The role of rare variants in determining the risk of psychiatric disorders has been studied, particularly for SCZ and other disorders with a relevant neurodevelopmental component. Whole exome sequencing (WES) studies demonstrated an enrichment in ultra‐rare variants in individuals with SCZ compared to controls, and specifically of loss of function (LoF) variants in LoF intolerant gene‐sets and highly brain‐expressed and evolutionarily constrained genes.^7^ However, studies using WES or whole genome sequencing (WGS) require large sample sizes to detect significant associations at the variant level, which are still lacking; therefore, previous studies have focused on testing the burden of variants in genes or groups of genes, using methods discussed in the next paragraph.

The importance of quantifying and understanding the genetic factors involved in the pathogenesis of psychiatric disorders has led to a substantial investment of resources in the development of ad hoc methods and in carrying out studies with increasingly large sample sizes. This has resulted in relevant advances in the field and the scope of the present review is to provide an overview and discussion of the methods, applications relevant to the clinic and possible future developments in psychiatric genetics.

## SELECTION OF RELEVANT STUDIES

This review summarizes the content of studies satisfying the following criteria: (1) focused on the identification of the genetic factors influencing the risk of psychiatric disorders or the outcomes of treatment with psychotropic drugs (efficacy/side‐effects); (2) based on the analysis or development of analysis methods for genome‐wide or next‐generation sequence data, or alternatively providing/discussing results with clinical applications in psychiatry; and (3) published in English until October 2021. This is a narrative review; therefore, it did not aim to provide a systematic assessment of all the studies in the field of interest.

## METHODS IN PSYCHIATRIC GENETICS

### Identification of variants, genes and gene‐sets associated with psychiatric disorders

The identification of variants and genomic regions or units that affect the risk of psychiatric illnesses is certainly one of the fundamental aims of research in psychiatric genetics. Two main types of studies can be used for this scope, namely GWASs and sequencing studies (WES or WGS). The former analyses common genetic variants only, while the latter includes rare genetic variants (a variant frequency of 1% in the population is often used as a threshold to distinguish between common and rare variants). Both types of study can test associations at variant, gene or gene‐set level, though sequencing studies have typically been underpowered to identify associations at the variant level, as noted in the Introduction.^7^

GWASs need large sample sizes to have adequate statistical power, particularly in the case of highly polygenic disorders, such as MDD, as they are characterized by the contribution of many variants with very small effect size.^8^ Relatively small sample sizes are likely to be another reason behind the previously mentioned missing heritability, as the increase in GWAS sample size is expected to increase the degree of genetic variance explained (the estimation of variant effects becomes closer and closer to the actual effect size). The sample size needed to identify SNPs that can explain 80% of GWAS heritability is between 0.7 and 1.5 million for most psychiatric diseases, but up to 10 million for MDD.^8^ However, it should be noted that the increase in GWAS sample size entails an increase in heterogeneity, which is a relevant issue for psychiatric disorders, and this may explain the lack of increase in SNP‐based heritability found in more recent and larger GWASs compared to older ones.^8^

Such large samples can only be collected within international consortia, and the Psychiatric Genomics Consortium (PGC) is one of the main groups involved in this effort. The approach adopted by the PGC consists in meta‐analyzing data from case–control GWASs available through world‐wide collaborations and, for example, it led to the inclusion of 306,011 individuals in recent SCZ GWASs^9^ (see Table 1 for an overview of recent GWASs of psychiatric disorders by the PGC). The progressive increase in sample size led to an increase in the number of genome‐wide significant loci, that is, the number of variants independently associated with a disease after multiple‐testing correction (Table 1).

**Table 1:** Overview of the most recent genome‐wide association studies by the Psychiatric Genomics Consortium (PGC)

Disorder	Sample size (cases/controls)	*N* of significant loci	SNP‐heritability (%)	Reference
Major depressive disorder	480,359 (135,458/344,901)	44	8.7	Wray et al.^10^
Schizophrenia	306,011 (69,369/236,642)	270	24	The Schizophrenia Working Group of the Psychiatric Genomics Consortium et al.^9^
Bipolar disorder	413,466 (41,917/371,549)	64	18.6	Mullins et al.^11^
Anxiety disorders	21,761 (7016/14,745)	1	9.5	Otowa et al.^12^
Obsessive–compulsive disorder	9725 (2688/7037)	0	28	International Obsessive Compulsive Disorder Foundation Genetics Collaborative (IOCDF‐GC) and OCD Collaborative Genetics Association Studies (OCGAS)^13^
Post‐traumatic stress disorder	206,655 (32,428/174,227)	3	5–20, varying by sex	Nievergelt et al.^14^
Anorexia nervosa	72,517 (16,992/55,525)	8	11–17*	Watson et al.^15^
Alcohol dependence	52,848 (14,904/37,944)	2	9	Walters et al.^16^
Cannabis use disorder	384,032 (20,916/363,116)	2	6.7–12*	Johnson et al.^17^
Opioid dependence	41,176 (4503/4173 opioid‐exposed controls and 32,500 opioid‐unexposed controls)	1	28 (cases vs. opioid‐unexposed controls)	Polimanti et al.^18^
Attention‐deficit hyperactivity disorder	55,374 (20,183/35,191)	12	21.6	Demontis et al.^19^
Autism spectrum disorders	46,350 (18,381/27,969)	5	11.8	Grove et al.^20^

*Estimation was reported for different population prevalences.

Other than individual loci, GWASs and sequencing studies offer the opportunity to test the joint effect of variants within a specific unit with biological relevance, such as a gene or gene‐set. This type of analysis has a higher power compared to variant‐level association tests and it also provides the possibility of clarifying the functional and biological mechanisms responsible for disease pathogenesis. In GWASs, one of the most used methods for this type of analysis performs gene analysis by projecting the matrix of variants in a gene into its principal components (PCs) to remove redundant parameters, then these PCs are used as predictors of the disease in a regression model. LD is accounted for and additional covariates can be included.^21^ Gene‐set analysis tests the joint effect of a group of genes that are related to each other, for example, genes coding for molecules that lead to a certain product or a change in the cell, such as a metabolic or signal transduction pathway, or are part of the same cellular structure. For gene‐set analysis, two main types of tests can be performed: a self‐contained or competitive test. The former tests the hypothesis that genes in a gene‐set have an effect on the phenotype significantly different from zero, while the latter tests the hypothesis that genes in a gene‐set are more strongly associated with the phenotype of interest than other genes (random pathway of the same size). Gene size, gene expression levels or other gene characteristics can be included in the analysis to evaluate if they affect the phenotype, considering conditional, joint or interaction effects.^21^

In WES/WGS studies, many tests have been developed to estimate the joint effect of variants in order to account for different hypotheses on the modality by which variants influence the phenotype. A common approach is represented by burden tests, which evaluate if a genetic score expressing the burden of variants in a genetic unit is associated with the phenotype, assuming all variant alleles have the same direction of effect on the risk of disease. This may be the case when variants are carefully selected for their probability of being causal (e.g., very rare variants, variants that modify the corresponding protein, such as LoF variants). However, when this assumption does not hold, the association signals of different variants may cancel out and lead to considerable loss of power. Alternatively, variance‐component tests consider the variance of genetic effects, or in other words the differences in the distribution of rare variants between cases and controls, assuming each variant may have a risk effect, protective or neutral effect. There is also the option to combine the two described approaches, as done in most studies, to account for both possible scenarios.^22^

In psychiatric genetics, gene and gene‐set analyses have yielded interesting results that indicate the involvement of processes regulating synaptic function, neuronal morphogenesis, neurodevelopment, neuronal excitability, and inflammation/immune response among others (Table 2).

**Table 2:** Overview of gene and gene‐set results found in PGC GWASs of psychiatric disorders

Disorder	*N* of associated genes	*N* of associated pathways and function	Reference
Major depressive disorder	*N* = 153 (many in the extended MHC region, others include *CACNA1E*, *CACNA2D1*, *DRD2*, *GRIK5*, *GRM5*, *PCLO*)	*N* = 19; RBFOX1, RBFOX2, RBFOX3, or CELF4 regulatory networks; genes whose mRNAs are bound by FMRP; synaptic genes; genes involved in neuronal morphogenesis; genes involved in neuron projection; genes associated with schizophrenia; genes involved in CNS neuron differentiation; genes encoding voltage‐gated calcium channels; genes involved in cytokine and immune response; genes known to bind to the retinoid X receptor	Wray et al.^10^
Schizophrenia	*N* = 130 (prioritized by fine mapping, 13 have synaptic annotations)	*N* = 27; neuronal excitability, development, and structure, with prominent enrichment at the synapse	The Schizophrenia Working Group of the Psychiatric Genomics Consortium et al.^9^
Bipolar disorder	*N* = 161 (15 prioritized by fine mapping, including *HTR6*, *MCHR1*, *DCLK3*, *FURIN*)	*N* = 4; synaptic signaling	Mullins et al.^11^
Anxiety disorders	*N* = 4 (*LOC152225*, *PREPL*, *CAMKMT*, *SLC3A1*)	/	Otowa et al.^12^
Obsessive–compulsive disorder	*N* = 4 (*KIT*, *GRID2*, *WDR7*, *ADCK1*)	*N* = 7; only gene‐sets associated with psychiatric disorders were examined, the top result was found for genes associated with schizophrenia	International Obsessive Compulsive Disorder Foundation Genetics Collaborative (IOCDF‐GC) and OCD Collaborative Genetics Association Studies (OCGAS)^13^
Post‐traumatic stress disorder	*N* = 12	*N* = 4; immune system, hypothalamic‐pituitary‐adrenal stress response, thioesterase binding	Nievergelt et al.^14^
Anorexia nervosa	*N* = 79 (including *NCAM1* and *CTNNB1*)	*N* = 1; embryonic development	Watson et al.^15^
Alcohol dependence	*N* = 0	/	Walters et al.^16^
Cannabis use disorder	*N* = 3 (*FOXP2*, *PDE4B*, *ENO4*)	*N* = 0	Johnson et al.^17^
Opioid dependence	*N* = 3 (*BEND4, C18orf32, SDCCAG8*)	*N* = 2; small RNA binding and genes downregulated 6 h after induction of HoxA5 expression	Polimanti et al.^18^
Attention‐deficit hyperactivity disorder	*N* = 20	*N* = 0	Demontis et al.^19^
Autism spectrum disorders	*N* = 15 (including *KCNN2* and *CRHRl*)	*N* = 0	Grove et al.^20^

*Note*: See Table 1 for information on sample size.

An increased frequency of copy number variants (CNVs), including rare LoF variants, has been reported in several psychiatric disorders, such as SCZ and attention‐deficit hyperactivity disorder (ADHD) and, to a less extent, in BP and MDD.^23^ For example, pathogenic deletions on chromosome 2p16.3 were associated with SCZ and appear to specifically disrupt the gene encoding the synaptic cell adhesion molecule neurexin‐1 (*NRXN1*). The effect sizes of CNVs of known psychiatric relevance far exceed those of common variants (e.g., odds ratio [OR] of 2–60); however, none is necessary or sufficient to determine the disease, or it is univocally associated with only one neuropsychiatric disorder, confirming the common pleiotropic effects of these variants.^23^

### Heritability and genetic correlation

An interesting area of research involves the estimation of heritability of psychiatric disorders and the genetic overlap across them. As mentioned at the end of the previous paragraph, there is strong evidence that the same variant can affect the risk of different psychiatric disorders (pleiotropy), and this has been explored extensively in GWASs. As discussed, however, GWASs focus on common variants only, and they estimate the proportion of heritability explained by SNPs (SNP‐based heritability or SNP‐based h2).

There are different methods to estimate SNP‐based h2 and genetic correlation (*r*_g_); some require raw genetic data (individual‐level genotypes), while others work on summary statistics (i.e., statistics describing the association of each variant with the phenotype, which typically include effect size, standard error, and *P*‐value). These methods can be also classified based on a model's assumptions and the method used to estimate SNPs effect sizes, including linear mixed models (LMM), Bayesian models, and LD‐adjusted kinships; a detailed review was recently published on the topic.^24^

Available software includes GCTA GREML (genome‐wide complex trait analysis, genome‐based restricted maximum likelihood) and the Bayesian variant GCTB, LDSC (LD score regression) and GEMMA (genome‐wide efficient mixed model association). LDSC and GEMMA can work on both quantitative and binary traits and accept summary statistics as input, while GCTA and GCTB require individual‐level genotypes.^24^ There is no optimal method/software, but each has advantages and limitations. For example, LMM assumes that all SNPs have nonzero effects with their effect sizes following a normal distribution, and this approach is more accurate in estimating SNP‐based h2 when actually a large proportion of SNPs have nonzero effects on the trait. On the other hand, when only a relatively small proportion of SNPs have nonzero effects, Bayesian variable selection regression (BVSR) tends to work better because it assumes that the genetic effect size of each SNP follows a point‐normal distribution. As genetic architecture is usually not known a priori, Bayesian sparse LMM can address this issue by combining the two distributions modelled in LMM and BVSR, as done by GEMMA. GCTA GREML is based on the LMM assumption, while GTCB implements a Bayesian approach to LMM, assuming that SNP effects are drawn from a mixture distribution of zero and nonzero components. Therefore, GCTB can estimate polygenicity, and another advantage is the estimation of the joint distribution of effect size and minor allele frequency, a useful indicator of negative selection.^25^ However, these methods do not take into account that variant effect size can be influenced by how many SNPs are in close LD, as done by others, such as LDSC. Instead of applying the standard likelihood‐based approach REML for fitting LMM, LDSC relies on a matching moments‐based method. GEMMA can also apply a variant of LMM using the method of moments, with a framework called MQS (MinQue for Summary statistics), which can effectively use a small random subset of individuals to produce unbiased and accurate estimates with calibrated standard errors.^24^ Even though simulations showed that LDSC estimates of SNP‐based h2 are generally biased downward, LDSC is a largely used software, because of a low computational burden and widespread availability of GWAS summary statistics.^3^ Overall, variability in SNP‐based h2 for a trait was demonstrated across different methods, but particularly across different samples, suggesting that heterogeneity across sampling populations is an important issue and that it may be partly responsible for missing heritability.^24^

GREML and LDSC methods both have bivariate extensions for the estimation of *r*_g_; notably, *r*_g_ is more robust to the assumptions made in different methods compared with SNP‐based h2 estimates, and *r*_g_ estimations show good concordance between family studies and GWASs for psychiatric disorders, suggesting that *r*_g_ estimated from common SNPs is the same as *r*_g_ estimated across the full allele frequency spectrum.^3^

Large GWASs of psychiatric traits estimated SNP‐based h2 of psychiatric disorders (Table 1) and demonstrated relevant genetic overlap across them (Table 3). For example, SCZ shows *r*_g_ of 0.72 with BP, 0.39 with MDD, 0.25 with anorexia nervosa, and 0.21 with ASD; among other disorders, MDD shows genetic overlap with BP (*r*_g_ = 0.38), ADHD (*r*_g_ = 0.42), and anxiety disorders (*r*_g_ = 0.80) (Table 3). Nonspecific psychiatric disorders heritability was demonstrated to show enrichment at regulatory chromatin active during fetal neurodevelopment; specifically, disruptions to synapse and calcium channel biology during neuronal proliferation, migration and establishment of circuits may play an important role.^27^ Polygenic risk scores (PRSs) are another approach to exploring the genetic overlap among different traits, as discussed in the section on PRSs below.

**Table 3:** Genetic overlap among psychiatric disorders estimated using genetic correlation

MDD	**0.39**	**0.38**	**0.80**	‐	**0.62**	**0.28**	**0.56**	**0.32**	**0.42**	**0.44**
	SCZ	**0.72**	**0.35**	‐	**0.34**	**0.25**	**0.36**	**0.31**	**0.12**	**0.21**
		BP	**0.26**	‐	0.18	**0.20**	0.19	**0.17**	**0.21**	**0.21**
			ANX	‐	‐	**0.25**	‐	‐	**0.34**	**‐**
				OCD	‐	**0.45**	−0.31	‐	‐	‐
					PTSD	‐	‐	**0.42**	0.60	0.04
						AN	0.03	0.006	**−0.24**	0.06
							ALCDEP	**0.55**	**0.44**	−0.08
								CUD	**0.53**	**‐**
									ADHD	**0.38**
										ASD

*Note*: References.^3^, ^10^, ^11^, ^14^, ^15^, ^16^, ^17^, ^19^, ^20^, ^26^: Significant genetic correlations are in bold. For some disease pairs, no data were available.

ADHD, attention‐deficit hyperactivity disorder; ALCDEP, alcohol dependence; AN, anorexia nervosa; ANX, anxiety disorders; ASD, autism spectrum disorders; BP, bipolar disorder; CUD, cannabis use disorder; MDD, major depressive disorder; OCD, obsessive–compulsive disorder; PTSD, post‐traumatic stress disorder; SCZ, schizophrenia.

### Identifying causal variants: Fine mapping

GWASs have identified hundreds of loci associated with psychiatric disorders over the past 15 years; however, these results per se do not indicate the true causal variants/genes implicated because they cannot distinguish a causal variant from other variants that are in LD with it. This is the aim of fine mapping methods, which include many biological and statistical approaches to discovering the underlying etiopathogenetic mechanisms of a disease.

Biological approaches to fine mapping consist of checking which variants in a locus have a functional role, for example, promoters and enhancers, using resources such as the database by the NIH Roadmap Epigenomics Mapping Consortium, which determined histone marks to locate functional elements in 127 different cell and tissue types.^28^ Regulatory regions can also be detected based on DNA accessibility, with methods such as DNase‐seq, or by identifying the inherent transcriptional activity of enhancers and promoters, with techniques such as GRO‐seq. The mentioned approaches have different sensitivities and accuracies in the mapping of active regulatory regions.^29^ Furthermore, the fact that a variant lies in a functional element does not necessarily imply that it results in a disruption of a biological process relevant to the disease of interest. Other methods detect other types of variant effects, for example variants that modify chromatin accessibility, cause alternative splicing that affects gene expression, disrupt transcription factor binding sites, or change the physical interactions of a regulatory region with its target genes (long‐range interaction).^29^ Integration of genetic data with gene expression data generates eQTL (expression quantitative trait locus) information, which provides likely associations between variants and gene expression level, usually considering cis‐eQTL effects. Examples of eQTL databases are the Genotype‐Tissue Expression project (GTEx), including 54 tissues, 13 of which are in the brain, and PsychENCODE, based on post‐mortem prefrontal cortex tissue.^30^

An important observation is that most databases of functional annotations and eQTL do not include extensive information on spatial and temporal characteristics of the brain, that is, different regions of the brain and different phases of neurodevelopment that are relevant to psychiatric disorders, and only approximately 50% of eQTLs are shared across tissues and cell types.^30^

Other than gene‐expression‐based eQTL, other QTL types can provide insights into the functional role of GWAS loci, such as DNA methylation and other epigenetic mechanisms of regulation. A fascinating but challenging aspect of epigenetics is its role in the cross talk between the genome and the environment, as genetic variants can predispose to disease through the modulation of sensitivity to environmental risk, which modifies gene expression through epigenetic regulation. There are also genetically dependent DNA methylation domains with strong developmental stability and evidence of involvement in the risk of psychiatric disorders. For example, in patients with SCZ, DNA methylation variation was associated with polygenic burden for SCZ.^31^

Statistical methods to fine mapping include earlier approaches that assume at most one variant per locus has a causal role, which set the framework for later methodological developments. These approaches use Bayesian statistics to compare the marginal likelihood of the data at each SNP in a locus under different prior distributions for its effect on the phenotype. However, there is evidence that risk loci for a disorder can include more than one causal variant, which lead to the development of Bayesian fine‐mapping methods that jointly model multiple causal variants in each locus.^32^ The available software uses different search strategies for causal variants; some can incorporate functional data through principled prior specification (e.g., PAINTOR and DAP) and some can be applied to summary statistics (e.g., FINEMAP and SuSiE).^32^

Approaches to fine mapping were applied in recent large GWASs of psychiatric traits. For example, the last GWAS of SCZ identified 270 risk loci and applied several techniques to prioritize these results through fine mapping, resulting in 130 genes, of which 114 are protein coding. In particular, the study prioritized genes containing at least one nonsynonymous (NS) or untranslated region (UTR) variant with a posterior probability of causality ≥0.1 (FINEMAP) or genes the entire credible set was annotated to. This led to the prioritization of 19 genes based on NS or UTR variants, such as *SLC39A8*, which mediates zinc and manganese uptake, the voltage gated calcium channel subunit *CACNA1I*, interferon regulatory factor 3 (*IRF3*), and *GRIN2A*, encoding a glutamatergic NMDA receptor subunit. Other genes were prioritized based on effects on gene expression (eQTLs), such as the neurodevelopmental disorder gene *RERE*, *ACE* encoding angiotensin converting enzyme, and *DCLK3* encoding a neuroprotective kinase (Table 2). Prioritized genes were enriched for genes expressed in the brain and that are relatively intolerant to LoF mutations.^9^ Despite the usefulness of the described methods to restrict the list of potential causal variants, causality has to be confirmed using wet‐lab protocols, such as multiple parallel reporter assays or CRISPR screens, which can evaluate the functional effects of multiple variants simultaneously.^32^

### Involvement of specific cell types

The connection of genetic results to cellular experiments has become an important area of research to understand the etiopathogenesis of psychiatric disorders. As previously mentioned, it is important to have cell‐specific information when evaluating variant functionality and their impact on gene expression. Enrichment of GWAS significant loci for expression in specific cell types (obtained by single‐cell RNA‐sequencing [scRNA‐seq]) can also provide information on potential therapeutic strategies, as antipsychotic medication targets were associated with the same cell types as for the SCZ GWAS results.^33^

GWAS SCZ‐associated loci were enriched in human cortical inhibitory interneurons and excitatory neurons from cerebral cortex and hippocampus (pyramidal and granule cells), suggesting a primary involvement of neural cells, without restriction to a circumscribed brain region, in line with the widespread range of symptoms observed in this disease.^9^ These results are consistent with scRNA‐seq performed in post‐mortem prefrontal cortex tissue of patients with SCZ.^34^ Of note, they are also in line with the glutamatergic hypothesis of SCZ, postulating that dysfunctional NMDA glutamate receptors on gamma aminobutyric acid (GABA) cortical interneurons lead to excessive mesolimbic dopaminergic activity (positive symptoms) and to a deficit at the level of the mesocortical pathway (negative symptoms).^35^ Experiments on single cells, including scRNA‐seq, can also be used to understand the downstream effects of one or more genetic variant(s), looking at gene expression networks, for inferring causality in specific cell types and under certain conditions.^36^ For example, a recent study applied CRISPR editing to achieve allelic conversion for prioritized SCZ risk variants when only one putative causal SNP was predicted (*FURIN* rs4702), or CRISPR activation/inhibition (CRISPRa/i) to manipulate endogenous gene expression at loci containing several causal SNPs (*SNAP91*, *TSNARE1*, *CLCN3*). CRISPR editing of *FURIN* showed large cell‐specific effects: ASCL1/DLX2‐GABAergic neurons and NFIB‐astrocytes showed similar changes in *FURIN* expression, which was decreased compared to the control condition. However, the same variant allele induced increased gene expression in neural progenitor cells, with effects on neural migration. These cell differences were not detected in post‐mortem studies, suggesting that they may have been diluted in studies of brain homogenate. The results of CRISPRa/i experiments confirmed the impact of SCZ‐eQTL genes on neuronal branching, synaptic puncta density, and synaptic activity. The perturbation of the four genes in the direction associated with SCZ risk led to differential expression of 1261 genes, which impacted co‐regulated downstream genes/proteins and showed enrichment in gene‐sets associated with psychiatric disorders, but also SCZ‐relevant drug classes, including antipsychotics. Considering genes more downregulated than expected, the study reported enrichment for pre‐ and postsynaptic gene‐sets, particularly those regulating the secretion of glutamate and other neurotransmitters, synaptic vesicle trafficking and postsynaptic glutamate receptor signaling. Genes more upregulated were correlated with disorder signatures, and included genes with known rare CNVs or nonsynonymous de novo mutations associated with SCZ, but also SCZ GWAS genes, therefore linking rare and common risk variants.^37^

CRISPR and scRNA‐seq studies that focus on psychiatric disorders are still scarce, particularly for disorders other than SCZ. However, as previously noted, there are interesting applications to drug discovery and identification of markers of drug response. For example, cell abnormalities reversed by a specific medication may indicate clinical efficacy, as suggested by previous studies on lithium response in BP.^38^

### Polygenic risk scores

PRSs are used to study the genetic overlap between psychiatric disorders or with nonpsychiatric traits, but also between specific symptom dimensions and other traits, to understand the possible mechanisms behind the heterogeneity of psychiatric illnesses. PRSs estimate the cumulative effect of common variants associated with a trait at the individual level; different methods can be used to calculate the contribution of each SNP, from the simple use of GWAS effect sizes to Bayesian approaches, and all methods take into account the relationship between variants (i.e., LD).^39^

PRSs were studied as predictors of psychiatric disorders by estimating SNP effect sizes in a GWAS and then calculating the PRS for the disorder in an independent case–control sample. Common parameters to evaluate the predictive performance include the area under the receiver operating characteristics curve (AUC), which expresses the probability of distinguishing a case from a control. The AUCs of PRS of psychiatric disorders were reported to be 82% for SCZ, 67% for BP, while only ~58% for MDD, and 54% for anxiety.^40^ However, a more recent work showed an AUC of ~73% for SCZ, while a very similar AUC (~59%) for MDD.^41^ These results indicate that PRSs are currently not suitable for guiding the diagnosis of psychiatric disorders. However, PRS may have clinical utility in high‐risk individuals, such as those at high risk of psychosis, or to estimate the risk of developing SCZ in patients with first‐episode psychosis. For example, in first‐episode psychosis, individuals in the top quintile of SCZ PRS have an approximately twofold increased risk of being subsequently diagnosed with SCZ,^42^ and this information may guide the planning of follow‐up and preventive interventions (e.g., screening and control of modifiable environmental risk factors). In high‐risk individuals, the AUC of SCZ PRS in predicting the 2‐year psychosis conversion was 65%, with an explained variance of 9.2%–12.3%. The same study showed that SCZ PRS added to a psychosis risk calculator (based on clinical variables) may slightly increase the predictive performance.^43^ It was also suggested that PRS may add predictive value to stratifying patients when clinical risk factors are not prominent.^44^

PRS may also be useful to stratify patients in terms of prognosis and response to treatments. For example, higher PRS for SCZ was associated with a more chronic illness course, with negative and disorganized symptom dimensions, but not with positive symptoms.^7^ Another recent study found that SCZ PRS is associated with inpatient psychiatric treatment and risk of aggressive behavior, but SCZ PRS did not improve prediction compared to clinical variables only.^45^ Higher SCZ PRS was also reported to increase the risk of poor treatment response in MDD, BP, and SCZ.^46^, ^47^, ^48^

Another interesting application of PRS consists in studying the genetic overlap of psychiatric disorders with nonpsychiatric traits, an approach that can provide key information on the mechanisms linking psychiatric illness with medical comorbidities. The high comorbidity of cardio‐metabolic and inflammatory diseases with psychiatric disorders is a major issue, as it is an important contributor to disability and increased mortality.^49^ The clinical use of PRS of cardio‐metabolic diseases is particularly promising because these are demonstrated to add value to clinical variables for disease prediction, especially for early‐onset coronary heart disease.^50^ PRS demonstrated that there is a genetic overlap between some psychiatric disorders (MDD and ADHD) and cardio‐metabolic traits, such as BMI, type II diabetes, and coronary heart disease.^51^, ^52^ On the other hand, there is an inverse association between the PRS of SCZ and obesity,^53^ similarly to what has been found for anorexia nervosa and obsessive–compulsive disorder.^54^ In contrast to the latter disorders, polygenic associations of SCZ with abnormal glucose metabolism, increased waist‐to‐hip ratio and visceral adiposity were reported, suggesting that patients with SCZ may be genetically predisposed to metabolic disorders,^55^ but not to high BMI, as previously noted.

The relationship between cardio‐metabolic traits and MDD has been the focus of extensive research. Higher BMI was found to have a causal role in MDD, with a genetically determined 1 standard deviation higher BMI associated with higher odds of MDD (OR = 1.18–1.26).^56^ Another recent study demonstrated that the effect is specifically mediated by body fat mass.^57^ PRSs of BMI, leptin and C‐reactive protein were associated with an increased risk of MDD with atypical neurovegetative symptoms (increased weight and hypersomnia), but not typical symptoms.^58^ MDD with atypical symptoms is considered as an immune‐metabolic subtype of depression because of its genetic and biomarker profile, including neuroendocrine alterations and dysfunctions of brain circuitries integrating homeostatic and mood regulatory responses.^59^ These findings exemplify how genetic research can provide precious information to clarify the biological underpinnings of psychiatric disorders, linking specific clinical manifestations with etiopathogenetic mechanisms, and providing implications for treatment choice.

## APPLICATIONS TO THE CLINIC

The complex (gene–environment interactions) and polygenic architecture of psychiatric traits represents an important challenge to the identification of genetic profiles that show predictive performance suitable for clinical use. As a consequence, genetic testing is currently not recommended for aiding the diagnosis of psychiatric disorders, but it is recommended for neurodegenerative and neurodevelopmental disorders that also have common psychiatric manifestations, such as Huntington disease, Fragile X syndrome, early‐onset Alzheimer's disease, and frontotemporal dementia.^60^ Genetic testing may be useful in other diseases to inform life planning and to estimate risk in relatives, for example, global developmental delay, intellectual disability, and ASD, for which a molecular diagnosis can be obtained in at least a quarter of cases.^60^

Most current clinical applications of genetics in psychiatry concern the prescription of treatment (pharmacogenetics). These applications are based on the results of candidate gene studies that investigated variants in genes coding for cytochrome P450 (CYP450) enzymes. CYP450 enzymes are responsible for the metabolism of the greatest part of psychotropic drugs, particularly antidepressants and antipsychotics, and variations in their level/functionality were associated with significant changes in drug metabolism.^44^ Corresponding dose adjustments were calculated, as well as recommendations for choosing or avoiding some medications, and they are described in prescribing guidelines, such as those curated by the Clinical Pharmacogenetics Implementation Consortium (CPIC) and the Dutch Pharmacogenetics Working Group (DPWG), as illustrated in a recent review.^61^ Even though there is no clear demonstration of when and to whom pharmacogenetic testing of CYP450 variants should be offered, the available evidence suggests that those who did not respond or tolerate at least one previous antidepressant or antipsychotic are more likely to benefit from testing.^61^ In addition to the mentioned clinical applications of pharmacogenetics, HLA‐A and HLA‐B testing prior to use of carbamazepine and oxcarbazepine are recommended, as carriers of HLA‐B*15:02 or HLA‐A*31:01 alleles should avoid these drugs for the risk of severe cutaneous adverse reactions.^62^ Clinical recommendations based on pharmacogenetic testing should be ideally provided through decision support systems to guarantee a standardized interpretation of results and application of the corresponding therapeutic indications.

The translation of GWAS results to the clinic remains an area of high interest, as the potential impact of genetic information that is not confined to a few pharmacokinetic genes but captures key regions throughout the genome. Even though there is currently no recommendation for clinical use, there are direct‐to‐consumer genotyping services as well as companies offering interpretation of results of genome‐wide genotyping, including calculation of PRS for psychiatric and other disorders. To facilitate a scientifically sound and up‐to‐date interpretation of genetic data, we mention impute.me, an opensource, nonprofit web tool that allows the uploading of genome‐wide individual‐level data, to run genotype imputation, to calculate and interpret PRS of many traits, but also to have information on variants modulating medication response, appearance and physical fitness, and rare variants.^63^

The use of artificial intelligence is a possible approach to disentangling the complexity of psychiatric disorders pathogenesis by identifying patterns from genome‐wide or other multidimensional biomarker data, or combinations of biomarkers with clinical variables. However, predictive models of complex phenotypes (e.g., treatment‐resistant depression) created using machine learning showed insufficient performance in independent samples.^64^ This is likely a consequence of the high heterogeneity observed among individuals with the same psychiatric disorder/trait; the first step to overcome this issue could be the identification of more homogeneous groups, defined using, for example, a specific biomarker, possibly in conjunction with a measurable clinical dimension. A successful implementation of this approach led to the identification of the MDD subgroup with atypical neurovegetative features, as previously discussed.

## DISCUSSION

Psychiatric disorders and response to psychotropic drugs have a genetic component, as discussed in this review. However, the multi‐determined and polygenic nature of these traits represent relevant obstacles to the identification of valid and reproducible genetic and genetic–clinical profiles with diagnostic or prognostic value. Strategies that can be pursued to overcome these obstacles include the analysis of samples providing adequate statistical power, the improvement of phenotypic classifications, and the combination of complementary biomarkers.

The recruitment of large samples represents a relevant issue, because of the resources and time needed. However, the creation of international consortia and the availability of alternative data‐collection procedures have led to a consistent increase in the size of samples included in genetic studies. Alternative data collection consists of, for example, electronic health records (EHR) and digital phenotyping, that is, information that can be linked to genetic data generated from samples available in nation‐wide biobanks, such as the UK Biobank and Estonian Biobank. These collect biological samples from volunteers from the general population to study the role of individual genetic susceptibility and exposure to external factors in the development of specific diseases.^65^ Other than through online questionnaires, phenotyping can be based on EHR linked to individual genetic data, including hospital and primary care records. EHR can be seen as a compromise between minimal phenotyping based on self‐reported information and detailed phenotyping based on specialists‐rated scales, as EHR are recorded by physicians who are often not specialists and without the use of structured and standardized interviews/scales. EHR can include only standard codes, for example, for diagnoses, procedures and prescriptions, such as those linked to the UK Biobank, or also unstructured text notes, such as for the US biobank “All of Us.”^66^ In the latter case, natural language processing for automated feature extraction was demonstrated to have a high accuracy for determining psychiatric phenotypes, which was better compared to the use of billing codes only.^67^ However, the availability of some information may be limited or incomplete in EHR, for example, family history, lifestyle, environmental exposures, and treatment compliance. These data may be at least partly collected using online surveys/questionnaires.

The identification of patient subgroups that are homogeneous for certain measurable clinical and biological features represents another approach to improving power in genetic studies. The study of specific symptom dimensions that have known physiopathological mechanisms led to interesting results, for example, in the discussed case of atypical neurovegetative symptoms of MDD. Other examples are psychotic symptoms in BP that are predicted by higher SCZ PRS and manic symptoms in SCZ that are associated with higher BP PRS,^68^ demonstrating that the same clinical domain has a shared genetic basis independent of categorical diagnosis, and can be used to obtain a dimensional classification useful for research and potentially lead to clinical applications. This type of approach can be very useful also for developing new treatments, as some symptoms of psychiatric illnesses are still difficult to target using the available pharmacological options, and treatment choice would benefit from a higher level of personalization based on the individual symptom profile. A recent study investigated drug repurposing for MDD subtypes that are associated with treatment‐resistant depression, namely MDD with the atypical symptom weight gain and MDD with anxious features.^69^ The study used genome‐wide genotypes to impute gene expression levels and identify case–control differences; then, gene expression profiles of MDD subtypes were compared to in vitro drug‐induced gene expression changes, based on the hypothesis that drugs inducing opposite expression patterns may have a therapeutic potential. The results suggested that compounds modulating heat shock proteins and compounds acting on metabolism may be promising for MDD with anxious features and MDD with weight gain, respectively.

Other important and difficult challenges for future studies include the analysis of data of increasingly high complexity and dimensionality and the extension of genetic studies to more ethnically diverse populations. Complexity will increase not only because of the increasing availability of DNA sequence data, but also because of the generation of other biomarkers at the omics level (e.g., transcriptomics, proteomics) and the need to integrate these with genomic data. Integration of different layers of omics has been done scarcely so far because of the difficulty in obtaining these data in well‐powered samples and the largely unknown modes of interaction across them. Most studies integrated transcriptomics with genomic data to identify eQTL, for example.^70^ Of note, the influence of environmental factors during the life span is another factor that modifies these interactions. Gene expression risk scores and proteomic risk scores can be calculated to estimate the individual differences at the gene expression and protein level, respectively, using the same principle for calculating PRS (i.e., a weighted sum of biomarkers associated with a condition of interest); this could account for the effects of environmental exposures, but, differently to PRS, would not have a lifetime value.

Finally, it should be noted that most previous genetic studies were performed in samples of European ancestry and the increase in ethnical diversity is one of the main priorities of future studies.^71^ Limited ethnical diversity is an obstacle to the identification of genetic variants that are present only in some populations and to the proper estimation of variant effect sizes across different populations, with the risk of disparities in access to the benefits of knowledge generated by genetic studies.

## AUTHOR CONTRIBUTION

Chiara Fabbri is the only author of this review.

## CONFLICT OF INTEREST

Chiara Fabbri was a speaker for Janssen.

## FUNDING STATEMENT

None.

## ETHICS APPROVAL STATEMENT

N/A.

## Data Availability

N/A.
